# Medical management of vascular anomalies of the head and neck

**DOI:** 10.1111/jop.13324

**Published:** 2022-08-04

**Authors:** Christine Léauté‐Labrèze

**Affiliations:** ^1^ Unité de Dermatologie Pédiatrique et Centre de Référence des Maladies Rares de la Peau d'Origine Génétique Hôpital Pellegrin‐Enfants, CHU de Bordeaux Bordeaux Cedex France

**Keywords:** betablockers, hemangioma, mTOR, PROS, vascular malformation

## Abstract

Depending on impairment, treatment of vascular anomalies is decided on a case‐by‐case basis in pluridisciplinary consultations. Interventional treatments, especially surgery and sclerotherapy, are usually partially efficient and management of patients with vascular anomalies increasingly involves the use of medical drugs. The most common vascular tumor is infantile hemangioma where first‐line medical treatment, when necessary, is propranolol. Kasabach–Merritt phenomenon is now largely treated with sirolimus whereas first‐line treatment of coagulation disorders associated with venous malformations is based on low‐molecular‐weight heparins or direct anticoagulants. Sirolimus is the standard treatment for painful inflammatory manifestations of low‐flow vascular malformations such capillary, venous, and lymphatic malformations that can occur singly or in combination but PIK3CA inhibitors, originally developed in oncology, have shown promising results in patients with PIK3CA‐related overgrowth spectrum. Currently, medical treatments are poorly developed for high‐flow malformations such as arteriovenous malformations. However, new research aimed at delineating the different arteriovenous malformations based on molecular findings has given new hope for future development of targeted therapies.

## 1 INTRODUCTION

Vascular anomalies are variable in aggressiveness depending on their type, size, and topography as well as the age of the patient. Some vascular anomalies are responsible for cosmetic discomfort, but others can be life‐threatening. In most cases, the management of head and neck vascular anomalies requires a multidisciplinary approach involving surgical and/or laser medical treatment and, in some cases, interventional radiology. Some drug treatments have a proven antiangiogenic effect leading to shrinkage of the vascular anomaly, whereas others are only useful for improvement of functional ability such as anticoagulants used for venous malformations (VMs) that are often complicated by thrombosis. The last decade has been marked by the use of highly efficacious beta‐blockers for infantile hemangiomas (IHs) and by the growing interest of mammalian target of rapamycin (mTOR) inhibitors in various aggressive vascular anomalies with slow‐flow and/or partial lymphatic differentiation. More recently, identification of the molecular mechanisms associated with vascular anomalies has led to optimism that targeted therapies can be developed.

## 2 MEDICAL THERAPIES WITH ANTIANGIOGENIC DEMONSTRATED EFFECT

### 2.1 Beta‐blockers

Since the discovery of the efficacy of propranolol in 2008,^1^ oral beta‐blockers have become the first‐line medical treatment for complicated IHs. However, the mode of action of beta‐blockers remains poorly understood.^2^ It has been suggested that propranolol targets several cell types in IH including stem cells, endothelial cells, and/or pericytes by means of β‐adrenergic receptor‐dependent and ‐independent mechanisms. A recent proteomic analysis showed that aquaporin‐1 (AQP1), a membrane water channel protein modulated in tumor cell migration and invasion, is a major driver of beta‐blocker antitumor response. In IH samples, AQP1 was found exclusively in the perivascular layer made of telocytes. Functional in vitro studies showed that AQP1‐positive telocytes play a critical role in IH response to propranolol.^3^

Propranolol is the only drug officially approved by both the U.S. Food and Drug Administration and the European Medicines Agency for the treatment of IHs (see Table 1 for guidelines) based on a pivotal randomized study published in 2015.^4^ Oral propranolol is dramatically effective in treating IHs: when treatment is given early, at the onset of the proliferative phase, the tumor growth is stopped. Propranolol works rapidly and is particularly effective for treatment of subglottic IHs^5^, ^6^ and periorbital IHs^7^ that are responsible for visual axis obstruction. The indication and initiation of propranolol must be performed in an experienced center with monitoring of blood pressure and heart rate 1 h and then 2 h after first the dose, and then treatment is given on an outpatient basis. The recommended dose of propranolol is 3 mg/kg/day in two divided doses. In clinical studies, complete or almost complete disappearance of the IH was observed in 60% of cases after 6 months of treatment^4^ and in up to 75% of cases if the treatment was given until 1 year of age.^8^ When stopping propranolol, a relapse of the IH was observed in 10% to 15% of cases, and most often the regrowth was moderate and responsive to a further course of propranolol if necessary.^9^, ^10^ Relapses are frequently observed in large facial IHs, and the standard of care is to maintain treatment until 1 year of age in these patients. A recent study on large facial IHs showed that the median length of propranolol treatment was 16 months with a median age at treatment cessation of 21 months, and this was extended to 25 months if the V3 segment was involved (i.e., location in the beard area).^10^ Unlike corticosteroids, resistance to treatment is rare and late treatment of IHs beyond the proliferative phase is also possible.^11^

**TABLE 1:** Guidelines for use of propranolol in infantile hemangiomas (IHs)

Oral propranolol in head and neck IHs
**Indications**
IH with functional consequences (visual axis obstruction, stridor, feeding difficulties)
Large segmental IH (including PHACE syndrome)
Localized IH at risk of permanent disfigurement (including ulceration)
**Prior therapy**
Search for contraindication: careful questioning and clinical examination
Routine echocardiography and electrocardiogram are not necessary if basic cardiologic examination is normal
Electrocardiogram and cardiologic visit required in case of bradycardia and/or arrythmia at auscultation
**Initiation and monitoring**
Treatment should be initiated only in clinical setting equipped and qualified for the safe and immediate management of any adverse event (e.g., bradycardia)
Initial dosage of 1 mg/kg/day bid the first week, then increase to 2 to 3 mg/kg/d the following weeks
Monitoring of 2 h after the first intake and at each dosage increase
Maintain 2 to 3 mg/kg/day, bid for 6 to 12 months
Monitor children monthly: clinical evaluation + pictures
End of treatment: tapering is not necessary
Parents should be informed of the risk of relapse (10% to 15% of cases)
**Expected side effects**
At each visit, parents should be educated concerning the risk of hypoglycemia and respiratory symptoms (wheezing)
To avoid hypoglycemia, be sure that the infant feeds regularly; in case of poor food intake temporarily stop propranolol; in case of wheezing, also temporarily stop propranolol
Minor side effects: nothing to do in case of cold hands or asymptomatic low diastolic blood pressure; for nightmares avoid giving the treatment after 5 pm and/or reduce the dosage

The acceptability and tolerance of propranolol is usually good in infants.^12^ The main side effects reported in infants are hypoglycemia in fasting situations, worsening of bronchospasm during outbreaks of bronchiolitis, and sleep disturbances. Bradycardia is more rarely observed, and asymptomatic hypotension and diarrhea are sometimes present at the start of treatment. Propranolol is also the first‐line medical treatment for PHACE syndrome,^13^ as short‐ and long‐term safety data have been found to be reasonable.^10^, ^13^

Other beta‐blockers including nadolol,^14^ acebutolol,^15^ and atenolol^16^ have worked successfully in small series of IH patients. To date, however, there is not sufficient data to conclude these beta‐blockers are superior, nor a have better tolerance, moreover none of these molecules have a marketing authorization for this indication. Topical beta‐blockers have been applied on IHs, especially timolol which has been formulated as eye drops. The first open studies were encouraging and showed a potential effect on superficial IH,^17^ however, a recent randomized study demonstrated a limited benefit of timolol in resolving IH when given during the early proliferative stage.^18^ The conclusion is that the value of topical beta‐blockers in the management of complicated IH forms remains limited. In addition, other studies have shown that timolol is transcutaneously absorbed and significative blood levels have been found in infants^19^ which could cause systemic side effects.

In other vascular tumors, such as congenital hemangiomas (rapidly involuting [RICH]/non‐involuting [NICH]/partially involuting [PICH]), kaposiform hemangioendothelioma, and tufted angiomas (TAs), interest of propranolol seems anecdotal,^20^ except in angiosarcomas in association with radiotherapy and/or chemotherapy.^21^ Propranolol is unnecessary in LMs and VMs.^22^ Concerning arteriovenous malformations (AVMs), propranolol may help to reduce flow and ameliorate patient comfort^23^ and encouraging results on epistaxis has been reported in hereditary telangiectasia.^24^

### 2.2 mTOR inhibitors

Sirolimus, also called rapamycin, was discovered in the 1970s as a substance produced by *Streptomyces hygroscopicus*. Sirolimus was initially considered an antibiotic, then later used as an immunosuppressant. Current guidelines for sirolimus use are described in Table 2. It acts by inhibiting mTOR, a serine/threonine kinase regulated by phosphoinositide 3‐kinase (PI3K) and Akt, key elements of many cellular processes such as proliferation, apoptosis, and angiogenesis. Sirolimus thus has antiproliferative, immunosuppressive, antiangiogenic, and antilymphangiogenic properties.^25^ Sirolimus is currently used in oncology, especially in angiolipomas and astrocytomas linked to tuberous sclerosis. Other mTOR inhibitors, also referred to as rapalogs, include everolimus, temsirolimus, and deforolimus.

**TABLE 2:** Guidelines for use of sirolimus in vascular anomalies

Oral sirolimus/rapamycin in head and neck vascular anomalies
**Indications**
KHE/TA complicated by Kasabach–Merritt phenomenon
Low‐flow vascular malformation: LM & VM
Exceptionally: IHs, High‐flow malformation
**Prior therapy**
Search for contraindication: active infections (teeth), cancer, anemia and/or leucopenia, liver insufficiency, peanut allergy (oral solution), pregnancy
Ensure that vaccinations are up‐to‐date
Do: Blood count with platelets, extensive coagulation workup, renal and hepatic functions, lipids and glycemia, and depending on the patient situation: beta‐hCG, HIV, and hepatitis serology, QuantiFERON (+ chest X‐ray)
**Initiation and monitoring**
Initial dose of 0.08 to 0.1 mg/kg/day or 1.6 mg/m^2^/day, one dose in adults and two doses in children at a fixed time
Residual concentration of sirolimus must be dosed after 2 weeks, then every month; threshold value of the residual concentration is estimated between 4 and 12 ng/ml
Monitor children monthly: clinical evaluation
Ensure a good tolerance of treatment every month: blood count, renal and hepatic functions, lipids and glycemia
In case of lymphopenia, *Pneumocystis* prophylaxis is advised
Warning: bioavailability of the product is low, and it interacts with many molecules (e.g., grapefruit juice, antifungals, clarithromycin, rifampicin)
**Expected side effects**
At each visit, patient should be educated concerning the risk of infections
Most common side effects: oral mucositis, acne, digestive disorders (abdominal pain, anorexia), headaches, and asthenia
Anemia with microcytosis, lymphopenia, hyperlipidemia, hyperglycemia
Rare cases of hypersensitivity pneumonitis, arterial hypertension, and induced lymphoedema

Abbreviations: Beta‐hCG, beta human chorionic gonadotropin; HIV, human immunodeficiency virus; IH, infantile hemangioma; KHE, kaposiform hemangioendothelioma; LM, lymphatic malformation; TA, tufted angioma; VM, venous malformation.

The first publication reporting the efficacy of sirolimus in vascular anomalies concerned a child with hypertrophic syndrome associated with a germline mutation of the PTEN gene.^26^ More than a hundred publications have followed, mainly on vascular tumors complicated by thrombocytopenia and/or the Kasabach–Merritt phenomenon,^27^ and low‐flow vascular malformations (LM and VM).^28^, ^29^, ^30^, ^31^ Recently, a randomized clinical trial showed that sirolimus treatment led to a decrease in LM volume as well as improvement of oozing and bleeding and an increased quality of life.^32^ In cases of combined LM/VM, sirolimus significantly reduced pain, oozing, and bleeding, however, benefits were much lower than for cases with VM alone.^32^ Sirolimus has not been as effective in the treatment of AVMs.^33^ Recently, sirolimus has been proposed for the treatment of individuals with Sturge–Weber syndrome that have impaired processing speed or a history of stroke‐like episodes.^34^ However, a randomized, placebo‐controlled trial is needed to confirm these potentially beneficial effects. For IH patients with resistance to propranolol, sirolimus has been successful in isolated case reports.^35^

Sirolimus has the advantage, compared to other mTOR inhibitors, of having an oral route of delivery (either tablets or oral solution). Bioavailability is low, and it interacts with many molecules, particularly antifungals. Before initiating treatment, it is imperative to ensure that vaccinations are up‐to‐date, and to screen for contraindications including active infections, hematological disorders (anemia and/or cytopenia), liver insufficiency, peanut allergy for the oral solution form, and hypersensitivity to sirolimus. In vascular anomalies, sirolimus is usually initiated at a dose of 0.08 to 0.1 mg/kg/day or 1.6 mg/m^2^/day and is taken in 1 dose in adults and in 2 doses in children at a fixed time. The half‐life of sirolimus is about 60 h in adults, but it is shortened in children and variable according to age. At the beginning of treatment, biological examinations must be carried out monthly to ensure good tolerance (blood count, platelets, renal and hepatic functions, cholesterol, triglycerides, and glycemia are monitored). The residual concentration of sirolimus must be dosed after approximately 15 days to allow dosage adjustment: the threshold value of the residual concentration should align with the use of sirolimus in renal transplantation and is estimated between 4 and 12 ng/ml.^30^, ^32^

The side effects commonly reported during treatment with sirolimus in vascular anomalies are rarely severe; the most common are oral mucositis, digestive disorders (abdominal pain, anorexia), headaches, and asthenia. Rare cases of hypersensitivity pneumonitis have been reported, as well as cases of arterial hypertension and induced lymphoedema, presumably by inhibition of lymphangiogenesis.^36^ Biological disturbances may also occur: anemia and cytopenia, which may require discontinuation of treatment; microcytosis, which occurs frequently (probably linked to the interaction of sirolimus with iron metabolism); and elevation of blood lipids and glucose, which is usually without clinical consequences. The duration of treatment of vascular anomalies with sirolimus is at least 6 months, but the time course is not codified and is currently discussed between doctors, parents, and child. In the event of prolonged treatment, some teams advise *Pneumocystis* prophylaxis (cotrimoxazole or pentamidine).

Topical sirolimus is not marketed,^37^ but it has been utilized in numerous studies as a cream, gel, or solution at variable concentrations (from 0.1% to 8%). In topical use, sirolimus is not found in the blood and tolerance is usually good, however, local side effects occur in almost a third of cases which include tingling or pruritus. The most frequent indication of topical sirolimus is angiofibromas linked to tuberous sclerosis (also known as Bourneville disease). Other indications are Kaposi's sarcoma and cutaneous lymphangiectasias of LMs.^37^

### 2.3 Interferon alpha 2a and 2b

Interferon alpha is an antiangiogenic agent that decreases the proliferation of endothelial cells. Indications are currently limited to severe and complicated IH or vascular tumors not responding to propranolol nor corticosteroids^38^ or LMs with osteolysis.^39^ The dosage varies from 1 to 3 million units/m^2^/day by subcutaneous injection and duration of the treatment varies between 6 and 12 months. Fever and muscle aches (flu‐like symptoms) are common side effects,^38^ especially early in treatment. Less common side effects include hepatic and hematologic toxicity, hypothyroidism, and depressive syndrome, and in children severe neurotoxicity with spastic diplegia and developmental delay have been reported.

### 2.4 Vincristine

Vincristine is an antiangiogenic agent that interferes with mitotic microtubules and induces apoptosis of tumor cells. Prior to 2008, it was indicated in severe complicated IH that was unresponsive to corticosteroids or in Kasabach–Merritt phenomenon.^40^ Treatment modality is weekly intravenous administration of 0.05 mg/kg or 1 mg/m^2^ for at least 15 weeks. The efficacy rate is unknown but therapeutic effect against the vascular tumor typically begins 3 weeks following treatment initiation. Side effects are not trivial and may include fatigue, alopecia, constipation, abdominal pain, transient jaw pain, peripheral neuropathy, hematologic toxicity, and inappropriate secretion of antidiuretic hormone.

### 2.5 Thalidomide and lenalidomide

Thalidomide and lenalidomide are potent immunosuppressive and antiangiogenic agents effective in the treatment of inflammatory diseases and various cancers (e.g., myeloma, lymphomas, and epithelioid hemangioendothelioma). Some specialized centers use thalidomide for AVMs with and reported effectiveness against pain and bleeding,^41^ however, this drug has severe side effects such as neuropathy and teratogenicity.

### 2.6 Targeted therapies

Recently, identification of specific genetic mutations in vascular anomaly patients has made it possible to develop targeted therapies,^41^ especially for PIK3CA‐related overgrowth spectrum (PROS) patients with mosaic mutations in the PIK3CA pathway. Although PIK3CA inhibitors were originally developed for treatment of breast cancer, an open clinical study with alpelisib carried out on patients with PROS showed promising results.^42^ This was followed by an international randomized study which is still ongoing. Alpelisib seems well tolerated in patients^42^, ^43^: after 6 months of therapy an improvement of about 50% in volume of complex malformation was observed, mainly on the lymphatic components^43^; and pain and inflammatory flares were reduced as well as superficial capillary malformations. For AVMs, several publications identified somatic mutations in *MAP2K1* ^41^, ^44^, ^45^ *responsible for* increased MEK1 activity and endothelial cell dysfunction suggesting that MEK inhibitors could target sporadic AVMs,^45^ however, research trials have shown inconsistent results and many side effects. A clinical study is underway with trametinib.

## 3 ADJUVANT THERAPIES SOMETIMES HELPFUL IN VASCULAR ANOMALIES

### 3.1 Anticoagulants

Before the sirolimus era, aspirin and ticlopidine were proposed as first‐line treatments for platelet trapping observed in Kasabach–Merritt phenomenon^46^ but are currently used much less. Aspirin is occasionally prescribed in children for localized vascular coagulation of venous anomalies with inconsistent results^47^ or as palliative treatment in TAs to reduce inflammatory effects.^48^

Extensive VMs and/or LMs are frequently complicated by localized vascular coagulation^46^, ^47^ and the prescription of an anticoagulant is determined on a case‐by‐case basis and depends upon an individual's symptoms and risk factors. If patients frequently experience pain secondary to thrombophlebitis then prophylactic or therapeutic anticoagulation, specifically with low‐molecular‐weight heparin (LMWH) or direct oral anticoagulants (DOACs) (dabigatran, rivaroxaban, apixaban, edoxaban), can be proposed to alleviate pain. LMWH is the recommended treatment in cases of localized vascular coagulation that have worsened after surgery or an interventional radiological procedure.

### 3.2 Steroids

For many years, corticosteroids have been the first‐line treatment for complicated IHs and are still used as second‐line therapy if beta‐blockers are contraindicated.^49^ Corticosteroids are less effective than beta‐blockers and responsible for many side effects such as high blood pressure, slowdown of growth, and opportunistic infections. In Kasabach–Merritt phenomenon and inflammatory outbreaks of LMs, corticosteroids have been gradually replaced by sirolimus.^27^, ^32^

### 3.3 Tranexamic acid

Tranexamic acid is an antifibrinolytic agent that helps stabilize the clot. It can be used topically for controlling bleeding in vascular anomalies such as congenital hemangiomas^50^ or superficial lymphangiectasias of the tongue, however, it should be used orally with caution as it can promote thrombosis.

## 4 MEDICAL MANAGEMENT STRATEGY

**FIGURE 1:**
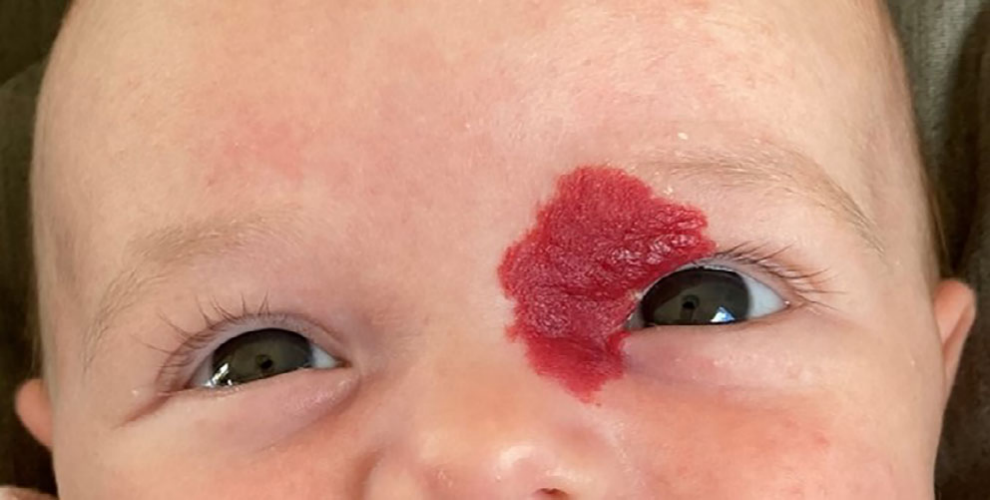
High risk infantile hemangioma at 2 months of age before treatment

**FIGURE 2:**
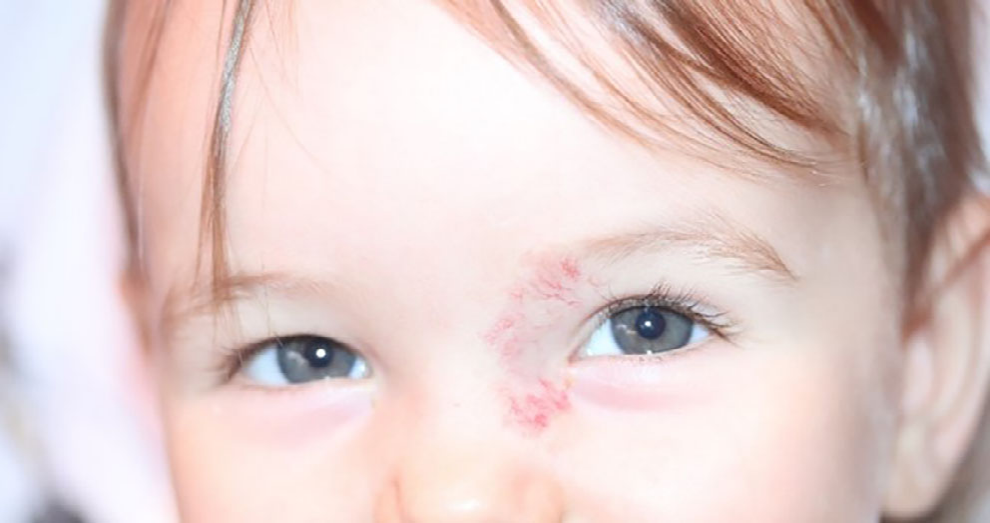
Same high‐risk infantile hemangioma at 12 months of age (10 months of propranolol therapy). No visual impairment is noted, if necessary, residual telangiectasia can be treated later with a vascular laser.

In practice, the drug treatment of AVMs (Table 3) is often integrated into a multimodal protocol that also includes interventional radiology, surgery, and/or laser therapy. The only situation where drug treatment may be sufficient is IH, since total or almost total disappearance of the lesion can be obtained in about 75% of cases with a beta‐blocker, most often propranolol. For at‐risk head and neck IH patients, drug treatment can save the child from invasive and painful procedures and propranolol has been shown to have the best risk/benefit ratio. Propranolol should be given as soon as possible to stop the IH from growing, to avoid functional complications, and to reduce long‐term esthetic consequences (Figures 1 and 2). In cases of contraindication to beta‐blockers or incomplete therapeutic result, surgery and/or laser treatment should be discussed for localized IH and drug treatments such as sirolimus should be considered for large IHs that are inaccessible to local treatment.

**TABLE 3:** Main treatments used for vascular anomalies

Infantile hemangiomas (IHs)	Propranolol^a^ (approved by the FDA and EMA) Atenolol^a^ Nadolol^a^ Acebutolol^b^ Topical timolol^a^ (mild efficacy) Corticosteroids^b^ Vincristine^c^ Interferon alpha^c^ Rapamycin^c^
Congenital hemangiomas (RICH/NICH/PICH)	Propranolol^c^ Rapamycin^c^
Kasabach–Merritt phenomenon (KHE/TA)	Rapamycin^b^ Corticosteroids^b^ Aspirin/ticlopidin^c^ Vincristine^c^ Interferon^c^ Propranolol^c^
Low‐flow malformations (capillary, LM & VM)	Rapamycin^a^ PIK3CA inhibitors^b^ (ongoing studies) Anticoagulants (aspirin, LMWH & DOACs) Tranexamic acid (topically applied)
High‐flow malformations (AVMs)	Propranolol^c^ Rapamycin^c^ Thalidomide^c^ MEK inhibitors^c^ (ongoing studies)

Abbreviations: AVM, arteriovenous malformation; DOAC, direct oral anticoagulant; EMA, European Medicines Administration; FDA, U.S. Food and Drug Administration; IH, infantile hemangioma; KHE, kaposiform hemangioendothelioma; LM, lymphatic malformation; LMWH, low‐molecular‐weight heparin; mTOR, mammalian target of rapamycin; NICH, non‐involuting congenital hemangioma; PICH, partially involuting congenital hemangioma; RICH, rapidly involuting congenital hemangioma; TA, tufted angioma; VM, venous malformation.

^a^ Efficacy proven by randomized studies.

^b^ Efficacy supported by open studies.

^c^ Efficacy supported only by small open studies or isolated clinical observations.

Congenital hemangiomas and vascular tumors that are complicated with Kasabach–Merritt phenomenon are very rare. Drug treatments are typically not effective on the tumor itself, however, sirolimus may be beneficial in the management of Kasabach–Merritt phenomenon.

Low‐flow VMs include capillary, venous, and lymphatic malformations that might also be found in combination. Interventional treatments, especially surgery and sclerotherapy, are usually only partially effective, and management of these patients increasingly includes medical drugs. Sirolimus is now a standard treatment in painful inflammatory manifestations of mixed and/or complicated LMs, but in the future it may be supplanted by more effective molecules targeting the PIK3CA/Akt pathway. Anticoagulants, aspirin, LMWH, or DOACs are often beneficial in patients with low‐flow malformations that are complicated by chronic localized coagulation and help to reduce thrombosis outbreaks and improve quality of life. Last, LMWH treatment is sometimes essential to avoid clotting imbalance after surgery or interventional radiological procedures.

For high‐flow malformations such as AVMs, no drug treatment has a proven efficacy against these potentially devastating disorders. Beta‐blockers or sirolimus can be used for palliative care to reduce flow and improve patient comfort. Currently, progress is being made in delineating the different types of AVM based on molecular findings which may lead to the development of targeted therapies. For example, a clinical study with the MEK inhibitor trametinib is currently being conducted. It is likely that in the future AVMs will be treated with a targeted drug therapy given before and/or after surgery or embolization similar to the way some cancers are now being managed.

## AUTHOR CONTRIBUTIONS

**Christine Leaute‐Labreze:** Conceptualization; writing – original draft; writing – review and editing.

## CONFLICT OF INTEREST

The author has declared that no competing interest exists as regards the conflict of interest.

### PEER REVIEW

The peer review history for this article is available at https://publons.com/publon/10.1111/jop.13324.

## Data Availability

The data that support the findings of this study are available from the corresponding author upon reasonable request.
